# Using Thermal Imaging to Monitor Body Temperature of Koalas (*Phascolarctos cinereus*) in A Zoo Setting

**DOI:** 10.3390/ani9121094

**Published:** 2019-12-06

**Authors:** Edward Narayan, Annabella Perakis, Will Meikle

**Affiliations:** 1 School of Agriculture and Food Sciences, Faculty of Science, University of Queensland, QLD 4072, Australia; 2 School of Science and Health, Western Sydney University, Penrith NSW 2751, Australia; 18024959@student.westernsydney.edu.au; 3 Wildlife Sydney Zoo, 1-5 Wheat Rd, Sydney NSW 2000, Australia; Will.Meikle@merlinentertainments.com.au

**Keywords:** thermal imaging, koalas, body temperature, heat/cold stress, thermoregulation, substrate, welfare, Zoo

## Abstract

**Simple Summary:**

Body temperature regulation is integral for the health and well-being of animals, especially in Zoo settings. Endothermic vertebrates such as small mammals are able to maintain a constant internal body temperature; however, extreme variation in body temperature may be reflective of underlying injuries or health issues. Thus, new technology that can enable the measurement of body temperature of small mammals without need for capture and handling can be very useful for the monitoring of animals in Zoos. In this study, we report the application of an IR thermal imaging camera for monitoring the body temperature of koalas. We found that the eye and abdomen were the most consistent body features to record body temperature. This tool will have useful application for welfare evaluation of small mammals, such as koalas in Zoos.

**Abstract:**

Non-invasive techniques can be applied for monitoring the physiology and behaviour of wildlife in Zoos to improve management and welfare. Thermal imaging technology has been used as a non-invasive technique to measure the body temperature of various domesticated and wildlife species. In this study, we evaluated the application of thermal imaging to measure the body temperature of koalas (*Phascolarctos cinereus*) in a Zoo environment. The aim of the study was to determine the body feature most suitable for recording a koala’s body temperature (using coefficient of variation scores). We used a FLIR530^TM^ IR thermal imaging camera to take images of each individual koala across three days in autumn 2018 at the Wildlife Sydney Zoo, Australia. Our results demonstrated that koalas had more than one reliable body feature for recording body temperature using the thermal imaging tool—the most reliable features were eyes and abdomen. This study provides first reported application of thermal imaging on an Australian native species in a Zoo and demonstrates its potential applicability as a humane/non-invasive technique for assessing the body temperature as an index of stress.

## 1. Introduction

Endothermic animals use various physiological and behavioural mechanisms to control body heat production and loss so that their internal body temperature remains constant, and this is considered “thermoregulation” [1]. The ability for an individual to thermoregulate allows them to cope with a range of environmental temperatures (i.e., extreme heat or cold), which could otherwise have detrimental effects on an animal’s well-being [2,3]. Specifically, “behavioural” thermoregulation depends on the spatial arrangement and availability of microclimates in an individual’s environment; most species can exploit cooler environments during hot weather and warmer environments during cold weather [4]. A suitable thermal landscape in wildlife enclosures is essential for captive management and animal welfare [5,6]. Zoo enclosures may have heating and cooling systems to provide suitable surrounding temperatures for animals [7], however it is often difficult to monitor body temperature of animals without handling. Recent technology advancements have allowed for thermal imaging to be a viable technique for measuring body temperature from a distance. An advantage of using thermal imaging is its high resolution, ability to contrast variation in body temperature, and non-invasive nature [8,9,10]. Thermal imaging has been used in veterinary medicine to detect leg and hoof problems using body temperature variation in racehorses, demonstrate high body temperature in livestock during transportation, and measure rapid changes in skin temperature in response to acute stressors [11,12]. To our knowledge, there is no published report on thermal imaging of native Australian wildlife in a Zoo setting.

Koalas are an arboreal folivore native to Australia [13] that use thermoregulation as a key survival mechanism to cope with subtle environmental changes, such as heat waves [3,4]. Koalas have an average body temperature between 35.5 °C and 36.8 °C and “behavioural” thermoregulation is important for maintenance of core body temperature [4,5]. Such behavioural thermoregulation in koalas includes panting to cool themselves, curling up into a ball to trap internal heat when cold, and the use of tree trunks as cold or warm substrates to regulate body temperature dependent on environmental conditions [4,5]. The selection of a perch by koalas has been demonstrated to be influenced by temperature, food availability, and time of day [4]. Interestingly, in response to temperature and “behavioural” thermoregulation, tree structure is an important consideration for koalas when selecting a perch, irrespective of food preference, because dense tree canopies provide a cool microclimate during hot weather [3,4,5]. Therefore, in koalas alone it is apparent that unsuitable microclimates and perch substrates in captivity can create difficulties to mimic natural thermoregulation and pose as potential stressors [14].

This preliminary study sought to record the body temperature of koalas using a thermal imaging camera to assess its applicability in a Zoo setting and specifically determine the most reliable body feature for taking body temperature readings. The application of this technology will help zookeepers to better understand the thermal preferences of koalas in relation to captive environments.

## 2. Materials and Methods

### 2.1. Study Population

Three captive-born and raised koalas at the Wildlife Sydney Zoo were observed: a 13-year-old female that has had multiple joeys (Erica), a 15-month-old juvenile male offspring of Erica (Birri), and a 16-month-old juvenile male (Alfie). The female koalas were socially housed in a large (62 m^2^) open enclosure consisting of seven tree branches. Juvenile male koalas are also socially housed (three koalas), but separately to the females, in smaller less exposed enclosures (23.4 m^2^) consisting of four branch perches.

### 2.2. Data Collection

**Figure 1:**
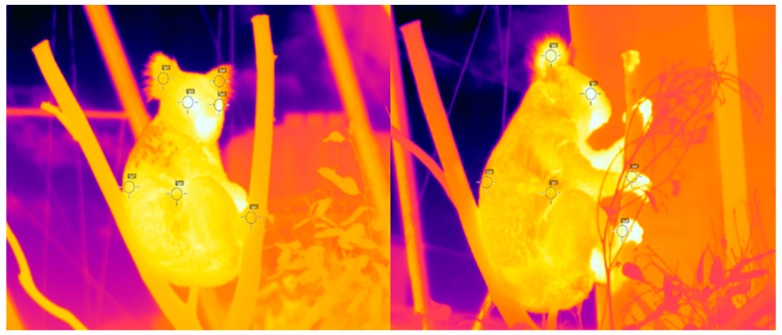
Thermal images of Erica on 14 (**left**) and 16 (**right**) May 2018 at Wildlife Sydney Zoo taken using a FLIR530 Thermal Imaging Camera. Both images show a minimally obstructed view of Erica perched on a branch, with the specifically selected body features highlighted by the black circular reticles.

All thermal images were taken using the FLIR530^TM^ Thermal Imaging Camera. Thermal images of each of the koalas were taken every 30 min between 8:00 and 17:00 (9 h a day) over three consecutive days in May 2018 for a total of 171 images (57 images per individual). Each image taken was focused and centred to the koala to incorporate as much of the body features within the image (Figure 1). Each image was taken within 1 m distance to the koala.

### 2.3. Image Analysis

All images were analysed using FLIR Tools version 5.13 software for extraction of thermal metrics. The FLIR Tools software enabled us to measure the temperature of specifically selected body features of a koala by simply defining them within each image (Figure 1). We were most interested in measuring the temperature of the eyes, ears, paws, abdomen, and back.

The coefficient of variation (the ratio of the standard deviation to the mean) was calculated for each body feature (eyes, ears, paws, abdomen, or back) to determine which was the most consistent for measuring body temperature using a thermal imaging camera, i.e., the body features with the least amount of variation.

## 3. Results

**Figure 2:**
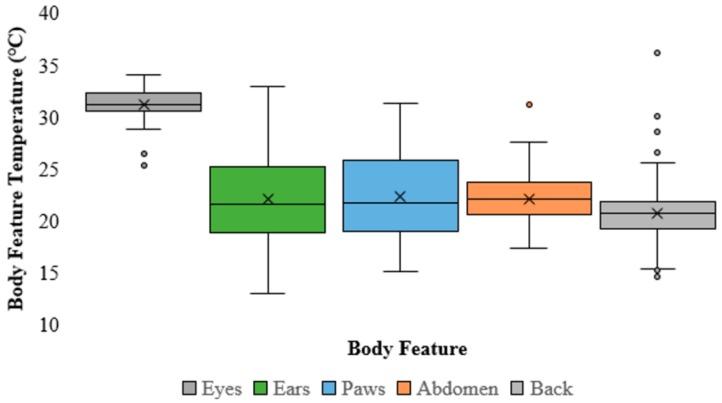
Boxplot demonstrating the range/spread of temperature (°C) for each body feature of a koala measured (from left to right: eyes; ears; paws; abdomen; back). The median (the line through each coloured box), average (“x”), and outliers (coloured circles) are shown for each body feature.

The eyes were the most consistent and hottest body feature for measuring the body temperature of koalas by a substantial margin—coefficient of variation: 4.83%, and average temperature: 31.16 °C (Figure 2). However, the eyes were one of the least available body features to measure being visible in only 40.35% of the images. The abdomen was the second most consistent body feature with a coefficient of variation of 11.01% (Figure 2) and was visible in 48.54% of the images. The back was the most available body feature to measure (69.60% of images) and was the third most consistent to measure body temperature (13.73%; Figure 2). The paws were the least available body feature to measure (39.18% of images) and were the fourth most consistent body feature (18.12%; Figure 2). The least consistent body feature to measure body temperature was the ears (19.68%; Figure 2), which were visible in 61.99% of images. For further details on the specific eyes, ears, or paws, refer to Table 1.

## 4. Discussion

Thermoregulation is the control of heat production and heat loss of an animal so that the internal body temperature remains at a more or less constant temperature; it is a property that most mammals such as koalas posses [12]. Specifically, koalas are endothermic marsupials, which have a thermal neutral zone (i.e., temperature tolerance) between 20 °C and 40 °C [1,2,3,4,5]; however, their average body temperature is regulated between 35.5 °C and 36.8 °C [1,3,4]. Our results from measuring these body temperatures using a thermal imaging camera (FLIR530^TM^) suggested that the eye was the most reliable external body feature for a koala. Using these measurements of body temperature and that of perch substrate we found that koalas use thermoregulation and thermal preference of substrates on which they were perched to regulate their body temperature (Figure 1 and Figure 2).

We were able to demonstrate in koalas that the eye was the most reliable external body feature for measuring body temperature. The lacrimal caruncle is small fleshy nodule located in the corner of the inner eye and is the hottest point of the eye, and thus the most representative external feature of core body temperature [10,15,16]. It is likely that this feature was reponsible for yielding eye temperature as our most accurate and representative reading of body temperature and that of previous studies [10,15]. Previously other studies have successfully used thermal imaging to measure core body temperature, including studies by [17], which confirms the use of eye temperature on unrestrained wild blue tits (*Cyanistes caeruleus*) as a non-invasive measure of physiological stress. Stewart et al. [18] further validates the uses of eye temperature by correlating core temperature in cattle as an indication for stress and pain. However, we could not confirm any correlation between our readings and core body temperature due to having no benchmark readings of core body temperature as measured by a tested and more invasive technique, such as a intraperitoneal device or rectal temperature reading [1]. Previous similar research [1] that did use a benchmark body temperature reading (intraperitoneal device) for comparison with various techniques demonstrated no correlation between thermal images and core body temperature in koalas. Nevertheless, [1] only used thermal images of the foot, a body feature which we have established as one of the least reliable, and thus future work investigating the correlation between thermal image readings of the eye and core body temperature in koalas could provide valuable insight. Johnson et al. [9] reported that from the thermal camera alone they were unable to give an accurate predication of core body temperature and were only able to give a correlation of it. We were able to validate the use of the eyes by not only measuring its temperature but that of other important body features for comparison of variation and repeatability. Of the three koalas measured for body temperature, all demonstrated a positive relationship between body temperature and perch substrate temperature, except, only Birri and Erica had a significant relationship.

### Study Limitations

Being able to identify a reliable body feature to measure the body temperature of an animal with a thermal camera introduces a non-invasive method for measuring animal welfare. Focusing on one body part reduces outliers and abnormally high readings (Birri’s max temp = 46.5 °C) that are also picked up by the thermal camera. The camera reads reflections and shadows that are present in the images therefore the camera is not reading the true temperature of the koala. This limitation has been found in similar studies, such as [10], where the solar radiation produced erratic temperature readings when reflected off other objects in the enclosure. This can been seen in the readings of the back and feet as there is such a high variation between the temperatures during the day for some koalas compared to another koalas in the same area. This variance is reflected in the CV%. The back and feet have the least consistent readings as koalas tend to sit in positions on the side of the trees or in branches which allows the sun to reflect off their backs. The nose of the koala was difficult to image because of the variability in positioning of the koala on the perches, hence it was not used in the image analysis. The camera was also picking up reflections off the artificial tree in the enclosure. Other limitations that are expressed in this study are distance from the thermal camera to the subject, as well as plants and other objects obstructing the analysed object.

## Figures and Tables

**Table 1:** Table displaying the sample size, average temperature (°C), and coefficient of variation for each of the five primary body features and the specific features that are entailed.

	Sample Size	Average Temperature (°C)	Coefficient of Variation (%)
Right Eye	45	31.27	4.36
Left Eye	42	30.63	8.65
Eyes	69	31.16	4.83
Right Ear	72	21.74	19.56
Left Ear	74	21.64	21.27
Ears	106	22.11	19.68
Right Front Paw	26	23.14	18.48
Left Front Paw	23	22.27	15.00
Right Back Paw	25	21.66	20.12
Left Back Paw	27	21.66	19.78
Front paws	43	22.55	16.25
Back Paws	47	21.48	20.07
Paws	67	22.36	18.12
Abdomen	83	22.13	11.01
Back	119	20.74	13.73
